# Supplementary material for: Impact of Traditional and Non-Traditional Lipid Parameters on Outcomes after Intravenous Thrombolysis in Acute Ischemic Stroke
Source: J Clin Med. 2022 Dec 1;11(23):7148. doi: 10.3390/jcm11237148 (PMC9737232; doi:10.3390/jcm11237148)
Supplement: Supplementary file 1 [file jcm-11-07148-s001.zip › jcm-2064216-supplementary.pdf]

Supplementary Material

Table S1: Baseline Characteristics of LAA vs non-LAA Stroke Mechanisms

	LAA (n=322)	Non-LAA (n=653)	*p*-value
Age (years)	65.00 [55.00, 76.00]	67.00 [57.00, 77.00]	0.143
Gender (male)	200/318 (62.9)	375/641 (58.5)	0.216
Race			0.338
Chinese	195/273 (71.4)	380/583 (65.2)	
Malay	49/273 (17.9)	129/583 (22.1)	
Indian	16/273 (5.9)	43/583 (7.4)	
Others	13/273 (4.8)	31/583 (5.3)	
Lipid parameters			
LDL-C (mmol/L)	2.91 [2.29, 3.69]	2.83 [2.12, 3.44]	0.027
HDL-C (mmol/L)	1.12 [0.94, 1.28]	1.12 [0.96, 1.34]	0.205
TC (mmol/L)	4.72 [3.93, 5.47]	4.60 [3.83, 5.33]	0.099
Non-HDL-C (mmol/L)	3.55 [2.81, 4.30]	3.42 [2.67, 4.13]	0.029
LDL-C/HDL-C Ratio	2.67 [1.93, 3.45]	2.40 [1.80, 3.22]	0.004
Stroke parameters			
Admitting NIHSS	16.00 [10.00, 21.00]	14.00 [7.00, 21.00]	0.001
Admitting SBP (mmHg)	152.00 [137.75, 169.00]	153.00 [136.00, 168.00]	1.000
Admitting DBP (mmHg)	83.00 [72.00, 91.00]	82.00 [73.00, 92.00]	0.898
Large vessel occlusion	236/292 (80.8)	348/598 (58.2)	<0.001
Comorbidities			
Smoker	49 (15.2)	97 (14.9)	0.957
Hypertension	207 (64.3)	437 (66.9)	0.456
Hyperlipidemia	168 (52.2)	348 (53.3)	0.794
Diabetes mellitus	90 (28.0)	207 (31.7)	0.262
Atrial fibrillation	1 (0.3)	189 (28.9)	<0.001
Glucose level			
Fasting glucose	5.90 [5.30, 7.43]	5.90 [5.20, 7.30]	0.586
HbA1c (%)	6.00 [5.70, 6.90]	6.00 [5.60, 6.90]	0.456
Biochemical parameters			
WBC (x10^9/L)	9.39 [7.63, 11.32]	8.30 [6.85, 10.17]	<0.001
Neutrophils (x10^9/L)	5.56 [4.43, 7.75]	4.87 [3.70, 6.80]	<0.001
Platelet (x10^9/L)	244.00 [201.00, 281.00]	232.00 [188.00, 274.00]	0.009

Values are median [IQR] for numerical variables and n/total (%) or n (%) for categorical variables. Abbreviations: LAA large-artery atherosclerosis, HbA1c Hemoglobin A1c, LDL-C high-density lipoprotein cholesterol, non-HDL-C non-high-density lipoprotein cholesterol, TC total cholesterol, HDL-C high-density lipoprotein cholesterol, WBC white blood cell, NIHSS National Institutes of Health Stroke Scale, SBP systolic blood pressure, DBP diastolic blood pressure

Table S2: Stroke Outcomes of LAA vs. non-LAA Stroke Mechanisms:

Stroke Outcomes	LAA (n=322)	Non-LAA (n=653)	*p*-value
Poor functional outcome (90-day mRS 3–6)	161/319 (50.5)	301/643 (46.8)	0.317
SICH	8/129 (6.2)	23/274 (8.4)	0.575
90-day mortality	39/320 (12.2)	71/645 (11.0)	0.663

Values are n/total (%). Abbreviations: LAA large-artery atherosclerosis, mRS modified Rankin Scale, SICH symptomatic intracranial hemorrhage.

Table S3: Baseline Characteristics of Study Population by LDL-C Quartiles

	LDL-C Q1	LDL-C Q2	LDL-C Q3	LDL-C Q4	*p*-value
n	254	249	253	248	
Age (years)	74.00 [63.00, 81.25]	67.00 [56.25, 77.00]	63.00 [54.00, 74.00]	62.00 [54.00, 72.25]	<0.001
Race					0.046
Chinese	161/222 (72.5)	147/221 (66.5)	144/218 (66.1)	134/222 (60.4)	
Malay	33/222 (14.9)	45/221 (20.4)	49/218 (22.5)	61/222 (27.5)	
Indian	17/222 (7.7)	14/221 (6.3)	11/218 (5.0)	20/222 (9.0)	
Others	11/222 (5.0)	15/221 (6.8)	14/218 (6.4)	7/222 (3.2)	
Gender (male)	142/251 (56.6)	142/247 (57.5)	150/243 (61.7)	155/245 (63.3)	0.357
Comorbidities					
Smoking	21 (8.3)	33 (13.3)	32 (12.6)	62 (25.0)	<0.001
Hypertension	175 (68.9)	167 (67.1)	163 (64.4)	152 (61.3)	0.303
Hyperlipidemia	139 (80.3)	125 (69.8)	113 (70.2)	149 (77.6)	0.052
Diabetes mellitus	85 (33.5)	72 (28.9)	63 (24.9)	86 (34.7)	0.068
Atrial fibrillation	62 (24.4)	59 (23.7)	37 (14.6)	32 (12.9)	0.001
Lipid Parameters					
LDL-C (mmol/L)	1.80 [1.45, 2.01]	2.52 [2.34, 2.69]	3.19 [3.02, 3.36]	4.08 [3.76, 4.66]	<0.001
HDL-C (mmol/L)	1.07 [0.90, 1.27]	1.12 [0.97, 1.33]	1.11 [0.94, 1.29]	1.18 [1.01, 1.34]	<0.001
Total cholesterol (mmol/L)	3.40 [2.95, 3.69]	4.25 [3.98, 4.51]	4.95 [4.70, 5.21]	5.97 [5.49, 6.54]	<0.001
Non-HDL-C (mmol/L)	2.30 [1.94, 2.53]	3.04 [2.82, 3.33]	3.77 [3.53, 4.05]	4.79 [4.32, 5.22]	<0.001
LDL-C/HDL-C Ratio	1.59 [1.31, 1.91]	2.23 [1.88, 2.58]	2.91 [2.40, 3.35]	3.61 [3.11, 4.13]	<0.001
Stroke parameters					
Admitting NIHSS	18.00 [10.00, 23.00]	15.00 [8.75, 21.00]	12.50 [7.00, 19.25]	13.00 [7.00, 20.00]	<0.001
NIHSS at 24h	9.00 [3.00, 19.00]	7.00 [2.00, 17.00]	5.50 [2.00, 13.00]	6.00 [3.00, 15.00]	0.003
Admitting SBP (mmHg)	152.00 [133.75, 167.00]	150.00 [135.00, 166.25]	153.00 [135.00, 168.00]	156.00 [140.00, 170.00]	0.077
Admitting DBP (mmHg)	81.00 [71.00, 91.00]	80.00 [72.00, 90.00]	84.00 [72.00, 92.00]	84.00 [75.50, 94.50]	0.017
Large vessel occlusion	180/239 (75.3)	154/219 (70.3)	122/232 (52.6)	140/226 (61.9)	<0.001
Onset-to-treatment Time (min)	155.00 [113.00, 213.00]	159.00 [120.25, 200.75]	153.50 [121.00, 202.00]	160.00 [126.75, 210.00]	0.658
Glucose level					
Fasting glucose (mmol/L)	6.00 [5.30, 7.10]	5.80 [5.23, 7.40]	5.90 [5.20, 7.10]	6.05 [5.20, 7.53]	0.657
HbA1c (%)	6.00 [5.60, 6.62]	5.90 [5.53, 6.60]	5.90 [5.60, 6.60]	6.10 [5.70, 7.90]	<0.001
TOAST					<0.001
Large-artery atherosclerosis	71/246 (28.9)	82/242 (33.9)	72/245 (29.4)	97/242 (40.1)	
Cardioembolic	117/246 (47.6)	93/242 (38.4)	70/245 (28.6)	61/242 (25.2)	
Small-vessel occlusion	31/246 (12.6)	35/242 (14.5)	57/245 (23.3)	45/242 (18.6)	
Stroke of other determined etiology	0/246 (0.0)	3/242 (1.2)	3/245 (1.2)	4/242 (1.7)	
Cryptogenic	27/246 (11.0)	29/242 (12.0)	43/245 (17.6)	35/242 (14.5)	

Values are median [IQR] for numerical variables & n (%) or n/total (%) for categorical variables. Abbreviations: Q1: first quartile, Q2: second quartile, Q3: third quartile and Q4: fourth quartile**.** LDL-C high-density lipoprotein cholesterol, non-HDL-C non-high-density lipoprotein cholesterol, TC total cholesterol, HDL-C high-density lipoprotein cholesterol. NIHSS National Institutes of Health Stroke Scale, SBP systolic blood pressure, DBP diastolic blood pressure, HbA1c Hemoglobin A1c. The quartiles for LDL-C were Q1: ≤2.18mmol/L; Q2: >2.18–2.86 mmol/L, Q3: >2.86–3.50 mmol/L, Q4: >3.50 mmol/L. The quartiles for non-HDL-C were Q1: ≤2.7mmol/L, Q2: >2.7–3.43 mmol/L, Q3: >3.43–4.19mmol/L, Q4: >4.19mmol/L. The quartiles for TC were Q1: ≤3.85mmol/L, Q2: >3.85–

4.63mmol/L, Q3: >4.63–5.36mmol/L, Q4: >5.36mmol/L. The quartiles for HDL-C were Q1: ≤0.95 mmol/L, Q2: >0.95–1.12 mmol/L, Q3: >1.12–1.32 mmol/L, Q4: >1.32 mmol/L. The quartiles for LDL/HDL ratio were Q1: ≤1.84, Q2: >1.84–2.49, Q3: >2.49–3.30, Q4: ≥3.30.

Table S4: Association of Lipid Parameters with Poor Functional Outcome, SICH and Mortality (Adjustment for Age and Gender only)

**Poor Functional Outcome (mRS 3–6)**			
	Multivariate Analysis		
	OR	95% CI	*p*-value
LDL-C			
Q1	1.42	0.97–2.09	0.072
Q2	1.0	-	-
Q3	0.89	0.61–1.31	0.563
Q4	1.01	0.69–1.48	0.953
Non-HDL-C			
Q1	1.79	1.21–2.65	0.004
Q2	1.34	0.91–1.97	0.134
Q3	1.0	-	-
Q4	1.29	0.88–1.91	0.194
TC			
Q1	1.85	1.25–2.74	0.002
Q2	1.43	0.98–2.10	0.067
Q3	1.0	-	-
Q4	1.24	0.84–1.83	0.276
HDL-C			
Q1	1.51	1.02–2.25	0.040
Q2	1.41	0.96–2.09	0.079
Q3	1.14	0.77–1.70	0.504
Q4	1.0	-	-
LDL-C/HDL-C Ratio			
Q1	1.60	1.08–2.36	0.018
Q2	1.68	1.14–2.47	0.009
Q3	1.0	-	-
Q4	1.88	1.27–2.78	0.002
**Symptomatic Intracranial Hemorrhage (SICH)**			
	Multivariate Analysis		
	OR	95% CI	p-value
LDL-C			
Q1	2.34	0.93–6.72	0.086
Q2	1.76	0.66–5.19	0.275
Q3	1.0	-	-
Q4	2.47	0.97–7.09	0.069
Non-HDL-C			
Q1	4.07	1.28–18.0	0.031
Q2	4.93	1.59–21.56	0.013
Q3	1.0	-	-
Q4	5.54	1.79–24.20	0.008
TC			
Q1	4.16	1.32–18.38	0.028
Q2	4.97	1.61–21.66	0.012
Q3	1.0	-	-

Q4	5.43	1.76–23.71	0.008
HDL-C			
Q1	1.0	-	-
Q2	2.75	1.12–7.75	0.037
Q3	1.72	0.62–5.15	0.304
Q4	2.23	0.86–6.51	0.113
LDL-C/HDL-C Ratio			
Q1	1.89	0.81–4.80	0.155
Q2	1.18	0.46–3.17	0.729
Q3	1.0	-	-
Q4	1.74	0.70–4.55	0.240
	**Mortality**		
	Multivariate Analysis		
	OR	95% CI	*p*-value
LDL-C			
Q1	2.56	1.40–4.95	0.003
Q2	2.06	1.09–4.06	0.030
Q3	1.0	-	-
Q4	1.59	0.79–3.25	0.195
Non-HDL-C			
Q1	1.54	0.86–2.87	0.155
Q2	1.72	0.95–3.19	0.078
Q3	1.0	-	-
Q4	1.08	0.55–2.12	0.829
TC			
Q1	1.78	1.01–3.24	0.051
Q2	1.49	0.82–2.76	0.197
Q3	1.0	-	-
Q4	1.04	0.53–2.02	0.916
HDL-C			
Q1	1.32	0.73–2.37	0.356
Q2	1.43	0.82–2.51	0.211
Q3	0.94	0.51–1.71	0.829
Q4	1.0	-	-
LDL-C/HDL-C Ratio			
Q1	1.61	0.89–2.98	0.124
Q2	1.90	1.06–3.51	0.035
Q3	1.0	-	-
Q4	1.28	0.65–2.52	0.480

Abbreviations: OR odds ratio, 95% CI 95% confidence interval, SICH symptomatic intracranial hemorrhage, mRS modified Rankin Scale, Q1: first quartile, Q2: second quartile, Q3: third quartile and Q4: fourth quartile. LDL-C high-density lipoprotein cholesterol, non-HDL-C non-high-density lipoprotein cholesterol, TC total cholesterol, HDL-C high-density lipoprotein cholesterol. Variables adjusted for in this multivariate analysis include age and gender only. The quartiles for non-HDL-C were Q1: ≤2.7mmol/L, Q2: >2.7–3.43 mmol/L, Q3: >3.43–4.19mmol/L, Q4: >4.19mmol/L. The quartiles for LDL-C were Q1: ≤2.18mmol/L; Q2: >2.18–2.86 mmol/L, Q3: >2.86–3.50 mmol/L, Q4: >3.50 mmol/L. The quartiles for TC were Q1: ≤3.85mmol/L, Q2: >3.85–4.63mmol/L, Q3: >4.63–5.36mmol/L, Q4: >5.36mmol/L. The quartiles for HDL-C were Q1: ≤0.95 mmol/L, Q2: >0.95–1.12 mmol/L, Q3: >1.12–1.32 mmol/L, Q4: >1.32 mmol/L. The quartiles for LDL/HDL ratio were Q1: ≤1.84, Q2: >1.84–2.49, Q3: >2.49–3.30, Q4: ≥3.30.

Table S5: Association of Lipid Parameters with Poor Functional Outcome, SICH and Mortality (Adjustment for Age, Gender, Admitting NIHSS and Large Vessel Occlusion).

**Poor Functional Outcome (mRS 3–6)**			
	Multivariate Analysis		
	OR	95% CI	*p*-value
LDL-C			
Q1	1.01	0.66–1.52	0.979
Q2	1.0	-	-
Q3	0.89	0.58–1.36	0.583
Q4	0.56	0.36–0.86	0.008
Non-HDL-C			
Q1	1.25	0.81–1.94	0.313
Q2	1.04	0.68–1.59	0.846
Q3	1.0	-	-
Q4	1.23	0.80–1.87	0.343
TC			
Q1	1.38	0.90–2.13	0.138
Q2	1.11	0.73–1.68	0.640
Q3	1.0	-	-
Q4	1.14	0.75–1.73	0.544
HDL-C			
Q1	1.80	1.17–2.78	0.008
Q2	1.79	1.17–2.75	0.008
Q3	1.59	1.03–2.45	0.037
Q4	1.0	-	-
LDL-C/HDL-C Ratio			
Q1	1.17	0.76–1.80	0.482
Q2	1.47	0.96–2.24	0.076
Q3	1.0	-	-
Q4	1.83	1.20–2.81	0.005
**Symptomatic Intracranial Hemorrhage (SICH)**			
	Multivariate Analysis		
	OR	95% CI	*p*-value
LDL-C			
Q1	0.51	0.17–1.42	0.205
Q2	1.49	0.67–3.48	0.338
Q3	1.0	-	-
Q4	1.06	0.46–2.56	0.887
Non-HDL-C			
Q1	3.13	0.97–13.97	0.083
Q2	4.14	1.32–18.26	0.028
Q3	1.0	-	-
Q4	5.33	1.70–23.46	0.010
TC			
Q1	3.36	1.05–14.94	0.064
Q2	4.10	1.32–18.01	0.028
Q3	1.0	-	-
Q4	5.14	1.65–22.55	0.011
HDL-C			
Q1	1.0	-	-

Q2	2.93	1.18–8.31	0.028
Q3	1.96	0.71–5.93	0.205
Q4	2.09	0.80–6.12	0.149
LDL-C/HDL-C Ratio			
Q1	1.49	0.63–3.81	0.378
Q2	1.07	0.41–2.88	0.889
Q3	1.0	-	-
Q4	1.67	0.66–4.43	0.284
	**Mortality**		
	Multivariate Analysis		
	OR	95% CI	*p*-value
LDL-C			
Q1	1.13	0.61–2.14	0.694
Q2	1.34	0.73–2.46	0.346
Q3	1.0	-	-
Q4	0.82	0.44–1.56	0.550
Non-HDL-C			
Q1	1.17	0.62–2.28	0.632
Q2	1.53	0.81–2.97	0.200
Q3	1.0	-	-
Q4	1.10	0.54–2.27	0.785
TC			
Q1	1.41	0.77–2.67	0.273
Q2	1.15	0.61–2.22	0.661
Q3	1.0	-	-
Q4	0.98	0.48–1.97	0.945
HDL-C			
Q1	1.37	0.74–2.55	0.310
Q2	1.62	0.91–2.93	0.106
Q3	1.21	0.64–2.28	0.550
Q4	1.0	-	-
LDL-C/HDL-C Ratio			
Q1	1.19	0.64–2.27	0.592
Q2	1.78	0.96–3.36	0.070
Q3	1.0	-	-
Q4	1.17	0.57–2.39	0.670

Abbreviations: NIHSS National Institutes of Health Stroke Scale, OR odds ratio, 95% CI 95% confidence interval, SICH symptomatic intracranial hemorrhage, mRS modified Rankin Scale, Q1: first quartile, Q2: second quartile, Q3: third quartile and Q4: fourth quartile. LDL-C high-density lipoprotein cholesterol, non-HDL-C non-high-density lipoprotein cholesterol, TC total cholesterol, HDL-C high-density lipoprotein cholesterol. Variables adjusted for in this multivariate analysis include age, gender, admitting National Institutes of Health Stroke Scale (NIHSS) and large vessel occlusion (LVO). The quartiles for non-HDL-C were Q1: ≤2.7mmol/L, Q2: >2.7–3.43 mmol/L, Q3: >3.43–4.19mmol/L, Q4: >4.19mmol/L. The quartiles for LDL-C were Q1: ≤2.18mmol/L; Q2: >2.18–2.86 mmol/L, Q3: >2.86–3.50 mmol/L, Q4: >3.50 mmol/L. The quartiles for TC were Q1: ≤3.85mmol/L, Q2: >3.85–4.63mmol/L, Q3: >4.63–5.36mmol/L, Q4: >5.36mmol/L. The quartiles for HDL-C were Q1: ≤0.95 mmol/L, Q2: >0.95–1.12 mmol/L, Q3: >1.12–1.32 mmol/L, Q4: >1.32 mmol/L. The quartiles for LDL/HDL ratio were Q1: ≤1.84, Q2: >1.84–2.49, Q3: >2.49–3.30, Q4: ≥3.30.
